# Flow regimes identification of air water counter current flow in vertical annulus using differential pressure signals and machine learning

**DOI:** 10.1038/s41598-024-63270-x

**Published:** 2024-05-31

**Authors:** Feng Cao, Ruirong Dang, Bo Dang, Huifeng Zheng, Anzhao Ji, Zhanjun Chen, Jiaxuan Zhao, Zhimeng Sun

**Affiliations:** 1 Xi’an Shiyou University, Xi’an, 710065 China; 2 PetroChina Changqing Oilfield Co., Xi’an, 710065 China; 3 Longdong University, Qingyang, 745300 China

**Keywords:** Chemical engineering, Natural gas, Fluid dynamics

## Abstract

This study investigates the gas–liquid two-phase counter-current flow through a vertical annulus, a phenomenon prevalent across numerous industrial fields. The presence of an inner pipe and varying degrees of eccentricity between the inner and outer pipes often blur the clear demarcation of flow regime boundaries. To address this, we designed a vertical annulus with adjustable eccentricity (outer and inner diameters of 125 mm and 75 mm, respectively). We conducted gas–liquid counter-current flow experiments under specific conditions: gas superficial velocity ranging from 0.06 to 5.04 m/s, liquid superficial velocity from 0.01 to 0.25 m/s, and five levels of eccentricity (e = 0, 0.25, 0.5, 0.75, 1). We collected differential pressure data at two distinct height distances (DP1: 50 mm and DP2: 1000 mm). We used vectors, composed of both the probability density functions (PDFs) of the differential pressure signals and the power spectral density (PSD) reduced via Principal Component Analysis, as features. Using the CFDP clustering algorithm—based on local density—we clustered the flow regimes of the experimental data, thereby achieving an objective and consistent identification of the flow regime of gas–liquid two-phase counter-current flow in a vertical annulus. Our analysis reveals that for DP1, the main differences in the PSD of various flow regimes occur within the 0.5–1 Hz range. Among the three flow regimes involved, the slug flow exhibits the highest power intensity, followed by the bubbly flow, with the churn flow having the least. In terms of differential pressure distribution, the bubbly and churn flows have a concentrated distribution, while the slug flow is more dispersed. For DP2, the PSD differences primarily exist within the 0.5–2 Hz range. The churn flow has the highest power intensity, followed by the slug flow, with the bubbly flow being the weakest. Here, the bubbly flow's differential pressure distribution is concentrated, while the slug and churn flows are more dispersed. Based on the results of the flow regime classification, we generated a flow regime map and analyzed the influence of annulus eccentricity on the flow regime. We found that in most cases, pipe eccentricity does not significantly affect the flow regime. However, in the transition region—such as the bubbly to slug flow transition zone—flows with medium eccentricity values (e = 0.5, 0.75) are more likely to transition to slug flow. We compared the visual recognition results of flow regimes with the clustering results. 4.04% of the total samples showed different results from visual recognition and clustering, primarily located in the flow regime transition area. Since visually distinguishing flow regimes in these areas is typically challenging, our methodology offers an objective classification approach for gas–liquid two-phase counter-current flow in a vertical annulus.

## Introduction

The gas–liquid two-phase counter-current flow in a vertical annulus is involved in numerous industrial fields such as petroleum, chemical, and nuclear energy. For instance, in the wellbore of coalbed methane vertical wells with liquid pump drainage, the formation-produced water flows downward in the annulus, and is expelled through the tubing by the pump. The formation-produced gas flows upward and is discharged from the wellhead. In the annular space formed by the tubing and casing, the region where the gas and liquid phases coexist is in a state of two-phase counter-current flow^1,2^, Accurately identifying the gas–liquid two-phase flow regime in the wellbore of coalbed methane wells is crucial for predicting the production dynamics of the coal seam^3,4^, However, the gas–liquid two-phase flow regime within the vertical annulus is controlled by various factors, such as the physical properties of the medium^5,6^, flow rate^7^, annulus structure^8–10^ etc. Among them, there is less literature on the structure of the annulus, especially the impact of the degree of eccentricity between the inner and outer pipes on the flow regime, which requires further research.

Currently, the main focus of research on gas–liquid two-phase flow in annular spaces is primarily on bubble morphology^8–10^. Liquid holdup/gas holdup^11,12^, Pressure gradient^10,13^, differential pressure fluctuation characteristics^14,15^, flow regime identification and conversion boundary^16–22^, flooding(counter-current flow)^23^ and other issues, Table 1 summarizes representative experimental studies related to gas–liquid two-phase flow in vertical annular spaces. Upon comparison, it is found that the identification of flow regimes is primarily based on the recognition of Taylor bubbles during the slug flow stage^24^, The annular structure has an impact on the characteristics of Taylor bubbles. For instance, Sadatomi et al.^8^ studied gas–liquid two-phase flow in vertical pipes with various specifications, including rectangular, triangular, annular, and circular cross-sections. They found that the rise speed of individual bubbles in still water varies in pipes with different cross-sectional geometries, and it is essentially positively correlated with the equi-periphery diameter of the pipe. Kelessidis and Dukler^9^ examined the shape of Taylor bubbles in concentric and semi-concentric (e = 0, 0.5) annular pipes. They found that the annular shape affects the shape and speed of Taylor bubbles. In semi-concentric annuli, Taylor bubbles are located on the side with a larger gap between the inner and outer pipes. Furthermore, the cross-sectional area ratio occupied by Taylor bubbles in semi-concentric annuli is relatively smaller than in fully concentric annuli. The comparison of Das et al.^25^ to different specifications of concentric annuli indicates that the rise speed of Taylor bubbles is solely a function of the annulus dimension. Caetano et al.^10^ compared the movement of Taylor bubbles in concentric and fully eccentric annuli (e = 0, 1). The results showed that the shape of Taylor bubbles differed in the two types of annuli. The movement of Taylor bubbles was more stable in fully eccentric annuli, and their rise speed in both types of annuli was higher than in a circular pipe with the same hydraulic radius.

**Table 1:** Summary of gas–liquid two-phase flow research in vertical annular spaces.

Researcher	Flow regime	Inner diameter of outer pipe (m)	Outer diameter of inner pipe (m)	Pipe length (m)	Flow direction	Eccentricity	Inclination	Fluids
Sadatomi et al.^8^	B,S,A	0.03	0.015	3.6	Co-current	0	Vertical	Natural gas/water
Kelessidis & Dukler^9,19^	B,S,C,A	0.0762	0.0508	6.93	Co-current	0,0.5	Vertical	Natural gas/water
Hasan & Kabir^18^	B,S,C,A	0.127	0.048, 0.057, 0.087	5.5	Co-current	0	vertical /inclined	Natural gas/water
Caetano et al.^10,13^	B,DB,S,C,A	0.0762	0.0422	13.7	Co-current	0,1	Vertical	Natural gas /water, Natural gas /kerosene
G. Das et al.^21,22,25^	B,DB,S,C	0.0508, 0.0381, 0.0254	0.0254, 0.0127, 0.0127	2.0	Co-current	0	Vertical	Air/Water
Takashi Hibiki et al.^11,12^	B,S,C,A	0.0381	0.0191	2.67	Co-current	0	Vertical	Water/steam
J.J. Jeong et al.^29^	B,CS,CT	0.0381	0.0191	4.37	Co-current	0	Vertical	Natural gas/water
Ozaret al.^30^	B,CS,C	0.0381	0.0191	4.37	Co-current	0	Vertical	Natural gas/water
Julia et al.^16^	B,CS,C,A	0.0381	0.0191	4.37	Co-current	0	Vertical	Natural gas/water
Julia et al.^17,31^	B,CS,C,A	0.0381	0.0191	4.37	Co-current	0	Vertical	Natural gas/water
Mendes^32^	B,DB,S,C,A	0.111	0.075	10.5	Co-current	0	vertical /inclined	Natural gas/water, Natural gas /oil
Colmanetti et al.^5^	B	0.15	0.06	10	Co-current	0	Vertical	Air/water/oil
Benjamin Wu et al.^23^	Homo,Hete,P–H,F	0.170,0.044	0.070,0.025	7.0,4.3	Counter-current	0	Vertical	Air/water
Colmanetti et al.^6^	B,DB,S,C	0.155	0.06	10	Co-current	0	0–90°	Natural gas /water/oil(3 viscosities)
Benjamin Wu et al.^20^	Homo,Hete, P–H,F	0.170	0.070	7.0	Counter-current	0	Vertical	Air/water

However, experimental results also indicated that due to Rayleigh–Taylor instability, slug flow is easier to identify in pipe columns with a hydraulic radius below 50 mm^26^ compared to those with a hydraulic radius above 100 mm^27,28^. The cross-section of the annulus greatly differs from that of the circular pipe, and its shape is related to the eccentricity. The formation mechanism of large bubbles in the annulus is more complex than in the circular pipe, thus bringing more uncertainties for visual recognition.

Due to the inherent difficulties in visually identifying two-phase flow regimes, various indirect detection methods have been developed, such as radiation^33,34^, conductivity^16,35–37^, fiber optic probe^34^, ultrasonic^38–40^, and differential pressure methods^15,41,42^. Among these, the differential pressure method has the advantages of being non-invasive, non-radioactive, and having a wide range of applications.

Due to the significant density difference between gas and liquid in two-phase flow, the volumetric ratio of gas bubbles significantly affects the average density, thereby influencing the hydrostatic pressure in the vertical direction of the pipe. The movement of bubbles causes fluctuations in the differential pressure between two points along the pipe's axis. Numerous studies have shown that the frequency and time domain features of the differential pressure signal are related to the two-phase flow regime^41,42^. For example, flow regime classification can be achieved based on the PSD of the differential pressure signal^20,38,43^, and the PDF based on differential pressure numerical interval statistics^15,41,44,45^.

Simultaneously, the distance of differential pressure signal collection is related to the two-phase flow characteristics it reflects^15,20^. Differential pressure signals collected at smaller distances (such as D/2) are more sensitive to smaller scale two-phase flow characteristics (such as individual bubbles and shorter Taylor bubbles). In contrast, those collected at larger distances are more sensitive to larger scale two-phase flow characteristics (such as pressure fluctuations in agitated flow). This study will use differential pressure signals collected at two different distances to obtain a more comprehensive reflection of two-phase flow characteristics.

Considering the above discussion, due to the complexity of gas–liquid counter-current flow in vertical annuli, its detection signal features have high dimensions. To avoid the subjective factors introduced in the manual flow regime identification process, many unsupervised machine learning classification methods have been applied in the field of gas–liquid two-phase flow regime recognition, such as K-means^34,46^, Fuzzy C -means^47^, self-organizing maps (SOM)^48^, expectation maximization (EM)^49^. These methods do not rely on prior information like flow regime labels, but solely on distance measures between experimental samples to classify flow regimes. The correspondence between classifications and flow regimes is then achieved by comparing the features of each classification. This study will be conducted based on this approach.

## Methods

### Experimental equipment and data acquisition

To study the problem of identifying flow regimes of gas–liquid two-phase counter-current flow in annuli at different eccentricities, a set of experimental pipe columns and auxiliary experimental equipment with continuously adjustable eccentricity was designed. The structure is described as follows:

The main body of the experimental pipe column is composed of two layers of acrylic pipes with a height of 6 m. The inner diameter of the outer pipe is 125 mm, and the outer diameter of the inner pipe is 75 mm. The inner pipe is sealed at both ends and only plays a role of occupying space, with the gas and liquid flowing in the annular space between the inner and outer pipes. An air inlet and a liquid outlet are set at the lower end of the outer pipe, and the upper end is connected to a gas–liquid separator to supply water to the pipe column and discharge gas.

**Figure 1:**
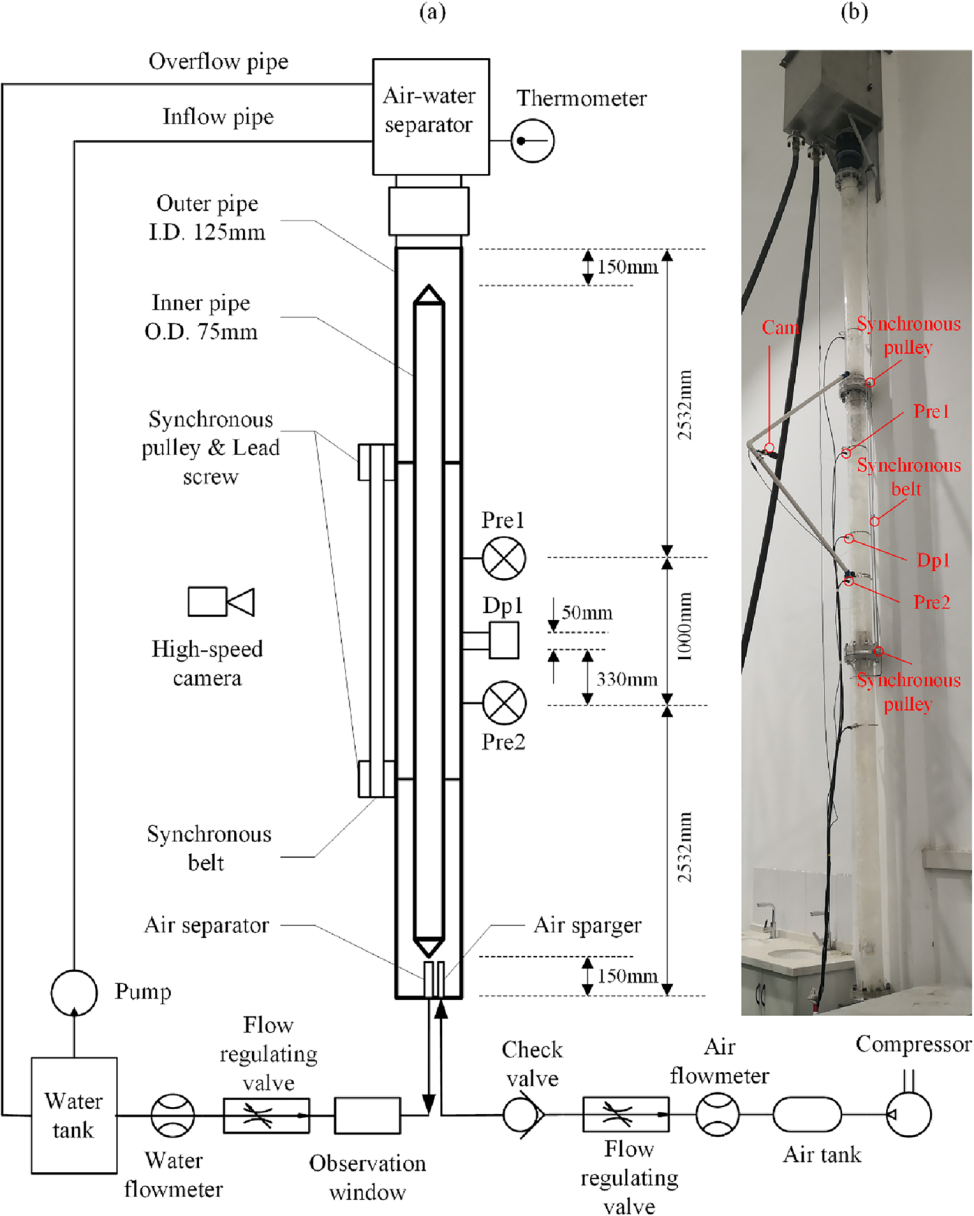
(**a**) Schematic diagram of the experimental apparatus and (**b**) Photograph of the experimental apparatus.

The experimental medium is water and air. During the experiment, the pump supplies liquid to the water tank, the water enters the pipe column from the water tank, and then is discharged through the liquid outlet and the flow rate is measured. The air enters the experimental pipe column from the bottom of the pipe column through the gas sparger after being measured in the pipeline from the compressor. After gas–liquid separation in the top water tank, it enters the atmosphere. Sensors for pressure, differential pressure, and temperature are installed on the pipe column. The overall structure of the system and the installation positions of the sensors are shown in Fig. 1.

The inner and outer pipes of the experimental pipe column are connected by two sets of T-shaped lead screws and slide sleeves at the 2.0 m and 4.0 m heights (from the bottom of the outer pipe). The slide sleeve is perpendicular to the pipeline axis and fixed on the inner pipe. The T-shaped lead screw pass through the slide sleeve and both ends pass through a flange ring clamped between the outer pipe flange joints, achieving a rotating seal. Therefore, by rotating the lead screws, the relative positions of the inner and outer pipes can be changed.

**Figure 2:**
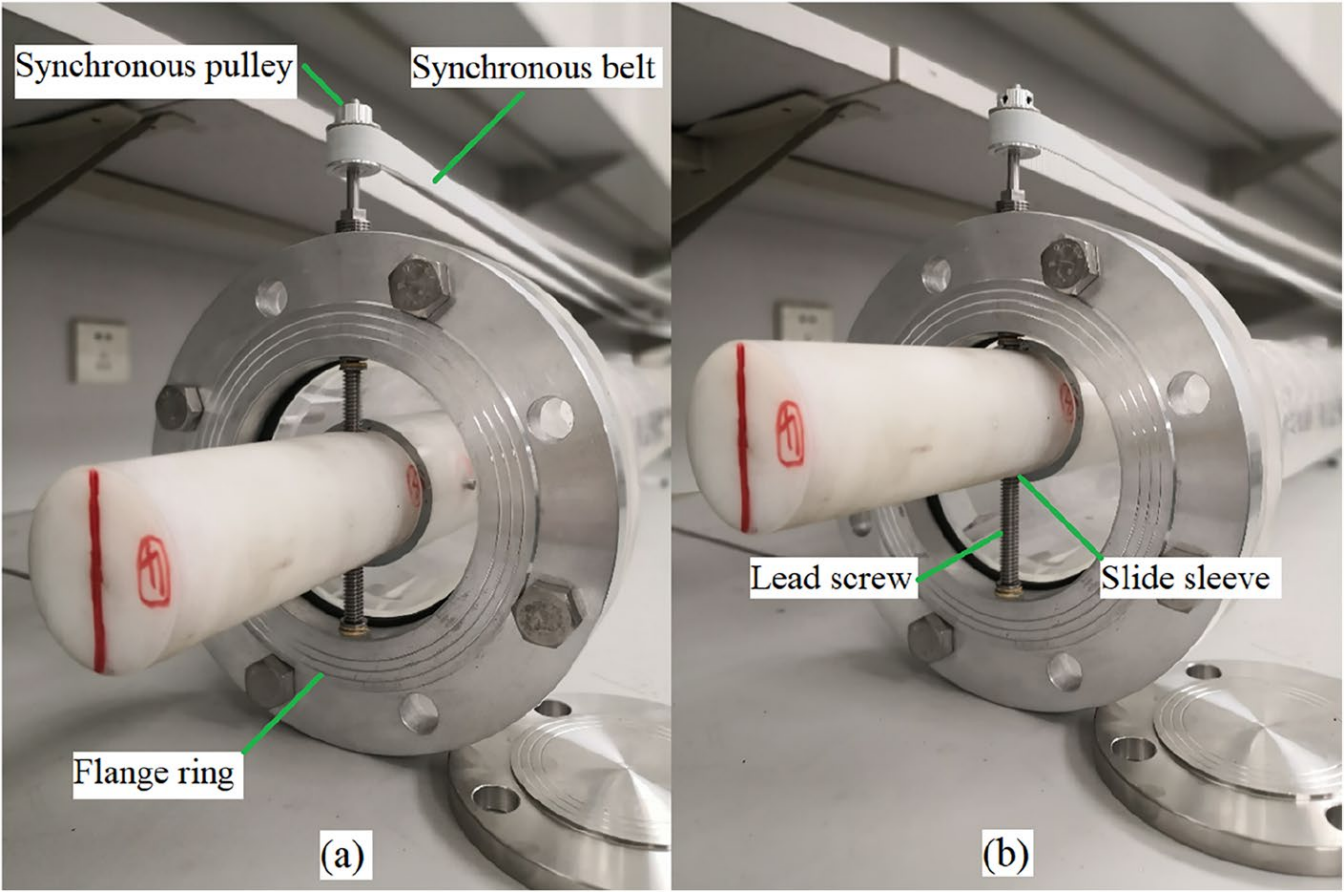
Eccentricity adjustment device. (**a**) e = 0, (**b**) e = 1.

The exposed ends of the lead screws are each fitted with a synchronous pulley. The synchronous pulley are connected by a synchronous belt, which can achieve synchronized rotation. Therefore, by pulling the synchronous belt, the inner pipe can be moved parallel to the outer pipe, achieving a continuous adjustment of the pipe column eccentricity within the range of 0–1, as shown in Fig. 2

The eccentricity is defined^10^ as the ratio of the distance between the axes of the inner and outer pipes to the difference in their diameter, as expressed in Eq. (1)

1 $$ e = \frac{{d_{oi} }}{{D_{o} - D_{i} }} $$

During the gas–liquid two phase counter-current flow, liquid flooding may occur when the liquid phase velocity is large, Sutton et al.^50^ conducted experimental studies on the entrainment phenomenon of downward flowing liquid on gas. Through experiments with air and water, they provided a boundary for liquid flooding, which occurs when the superficial velocity of the liquid phase exceeds 0.152 m/s. Further experimental research by Wu et al.^23^ pointed out that the flooding phenomenon of the gas and liquid phase may occur simultaneously in local areas, and the boundary for liquid flooding may be lower.

To prevent gas from entering the liquid discharge pipe and affecting the measurement of the liquid phase, a cylindrical gas separator densely covered with small holes was designed at the inlet of the liquid discharge pipe to prevent bubbles from entering. Through theoretical calculations and experimental verification, it can guarantee that no large bubbles will enter the liquid discharge pipeline when the superficial velocity of the liquid phase does not exceed 0.28 m/sec.

Experiments were conducted on the above experimental system under different combinations of gas, liquid flow rates, and eccentricities. All experiments were conducted under room temperature conditions (22 °C). Multiple flow combinations were selected within the range of gas superficial velocity Vsg = 0.06–5.04 m/s (standard conditions) and liquid superficial velocity Vsl = 0.01–0.25 m/s. Each flow combination was tested five times under e = 0, 0.25, 0.5, 0.75, 1.0 conditions, and eventually 495 groups of usable experimental data were obtained after data cleaning.

The Dp1 signal is collected by a differential pressure sensor with a measurement range of − 2.5–+ 2.5KP and an error of 0.1%. The Dp2 signal is obtained by subtracting the data synchronously collected by pressure sensors Pre1 and Pre2, both of which have a range of 20kPa and an error of 0.5%. All pressure taps are vertically arranged on the outer pipe wall, with their projection points coinciding on the annular cross-section. The distance between the Dp1 pressure taps is 50 mm, and the distance between the Pre1 and Pre2 pressure taps is 1000 mm, as shown in Fig. 1.

The liquid phase flow meter has a range of 1–10 m^3^/h with an error of 0.5%. The gas phase flow meter I has a range of 1–12 m^3^/h with an error of 1%, and the gas phase flow meter II has a range of 10–150 m^3^/h with an error of 1.5%.

The sampling rate of the sensor signal was 100sps with a sampling duration of 75s. All signals were transmitted using a 4–20 mA method. The transmission cable used a 0.5 mm^2^ shielded twisted pair, the sampling resistance was 150Ω with an accuracy of 0.01%, and the data acquisition card was a National Instruments USB-6361. All frequency converter input and output ends were equipped with filtering devices to eliminate electromagnetic interference from the frequency converter.

### Feature extraction

Before applying clustering methods to study flow regime classification, the raw data collected by the sensors underwent data cleaning, feature extraction, and normalization.

The differences in the differential pressure signals between different flow regimes of gas–liquid pipe flow can be quantified by their PSD and PDF. This paper uses the dimension-reduced PSD and PDF as features to characterize the distance measure of different flow regimes.

The work of several researchers has shown that there is a correlation between the features of the PSD of differential pressure fluctuations^15,44^ and the state of gas–liquid two-phase flow. However, the fluctuation features of different flow regimes differ only in certain frequency bands. Drahos̆ and C̆ermák^51^ as well as Letzel et al.^52^ pointed out that in circular pipes and bubble columns, the contribution of pressure fluctuations caused by bubble movement to the differential pressure spectrum is mainly concentrated in the 1–10 Hz range. Jaiboon et al.^43^ pointed out that the "slug" structure forms a peak in the 1.5–2 Hz area on the differential pressure signal spectrum. Benjamin Wu et al.^23^ found that individual small bubbles appearing from the air sparger are related to the peak near 11Hz on the differential pressure spectrum, and the flow of liquid also has an impact on this peak.

To highlight the main differences in pressure fluctuations under different flow regimes on the spectrum, the PCA technique was used to perform dimensionality reduction on the PSD. Before using the PCA technique, it is necessary to determine the Explained Variance Ratio (EVR) to be retained. Its value determines the data dimension after dimension reduction. The number of statistical intervals bins of the PDF also needs to be determined. During the research process, with the dataset shuffle seed and other algorithm parameters fixed, experiments were conducted at several levels of EVR at 0.8, 0.85, 0.9, 0.95 and bins at 50, 75, 100, 125, 150. The method is to gradually increase the EVR or bins until the average distance of the cluster centers no longer increases rapidly, that is, the value at the inflection point of the gain increase rate is chosen.

**Figure 3:**
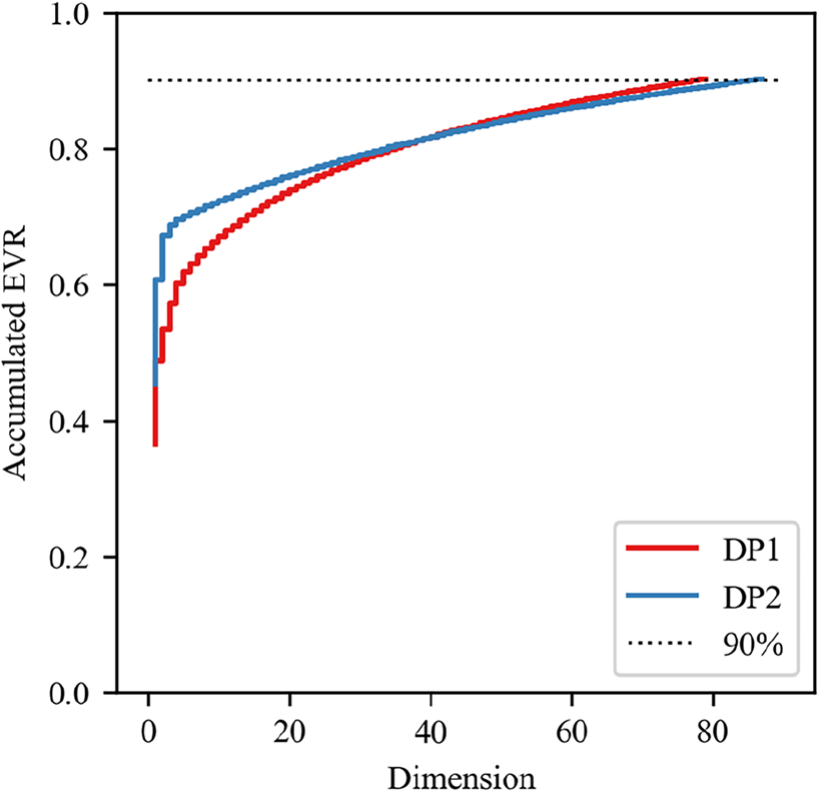
The relationship between the number of dimensions retained after PCA dimensionality reduction of the PSD of DP1 and DP2 signals, and their cumulative EVR.

The final determined EVR value is 0.9, which reduces the dimensions of the PSD of the DP1 and DP2 signals from 376 dimensions (corresponding to a sampling duration of 75 s and a sampling rate of 100 sps) to 79 and 87 dimensions, as shown in Fig. 3.

**Figure 4:**
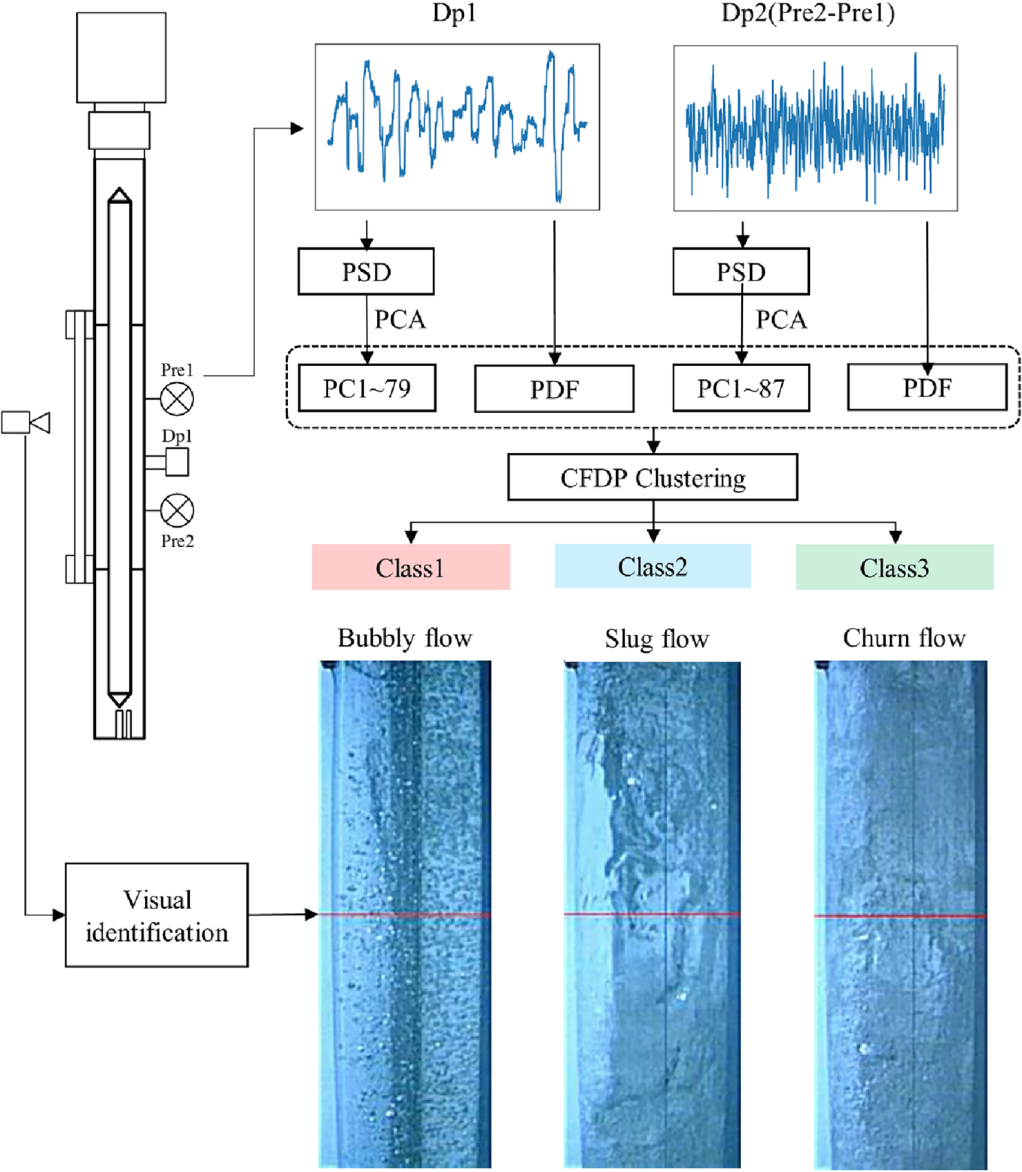
The flow regime classification process based on dual differential pressure signals and unsupervised machine learning methods.

The number of statistical intervals for the PDF is determined to be bins = 100. Therefore, the dimension-reduced PSD (79 dimensions + 87 dimensions) and PDF (100 dimensions + 100 dimensions), a total of 366 dimensions, are used as the distance measure between test samples. After normalization, this forms the dataset for subsequent machine learning. The overall process is shown in Fig. 4.

### Clustering

To avoid the subjectivity of flow regime classification through visual imaging, this paper uses an unsupervised machine learning method, namely clustering, to cluster the flow regimes involved in annular gas–liquid two-phase flow, in order to obtain a consistent and objective definition and description of flow regime characteristics.

In the past several decades, numerous clustering methods have been developed by different scholars^53^. As discussed in the first part, some classical methods have been applied to multiphase flow regime recognition problems. However, these clustering methods have two limitations: firstly, these classification algorithms make preliminary assumptions about the distribution structure of samples in the feature space. For example, the k-means algorithm assumes that the distribution of the same category in the feature space is spherical, which may not necessarily be consistent with reality. Secondly, these classification algorithms require manual specification of some hyper-parameter values, the selection of which has a significant impact on the classification results. For example, the number of classifications corresponding to the number of flow regimes, the artificially specified results are difficult to reflect the inherent distribution characteristics between categories.

To avoid the shortcomings of the above clustering methods, in this study, a clustering method based on p-density peaks, "Clustering by Fast Search and Find of Density Peaks (CFDP)", was used. It was proposed by Alex Rodriguez and Alessandro Laio^54^. Its basic assumption is that the class center is surrounded by neighbors with lower local density and is far away from those nodes with higher local density. The local density and distance are defined as follows:

2 $$ \rho_{i} = \sum\limits_{j} {\chi \left( {d_{ij} - d_{c} } \right)} $$

3 $$ \chi (x) = \left\{ {\begin{array}{*{20}c} {1,} & {if\; \, x < 0} \\ {0,} & {otherwise} \\ \end{array} } \right. $$

The distance $\delta_{i}$ from the class center to a node with higher local density is defined as:

4 $$ \delta_{i} = \mathop {\min }\limits_{{j:\rho_{j} > \rho_{i} }} (d_{ij} ) $$

For the point with the highest local density, take:

5 $$ \delta_{i} = \mathop {\max }\limits_{j} (d_{ij} ) $$

Determination of the cluster center. One of the advantages of this algorithm is the determination of the cluster center. After drawing the design map, the number of cluster centers with larger $\rho$ and $\delta$ values will be intuitively displayed.

In the case of a smaller dataset, the above algorithm can use the Gaussian kernel function to replace the truncated kernel function in Eqs. (2) and (3) to enhance the robustness of the algorithm. This paper uses formula (6) to calculate the local density, defined as follows:

6 $$ \rho_{i} = \sum\limits_{j \ne i} {\exp \left[ { - \left( {\frac{{d_{ij} }}{{d_{c} }}} \right)^{2} } \right]} $$

## Results and discussion

### Clustering results analysis

**Figure 5:**
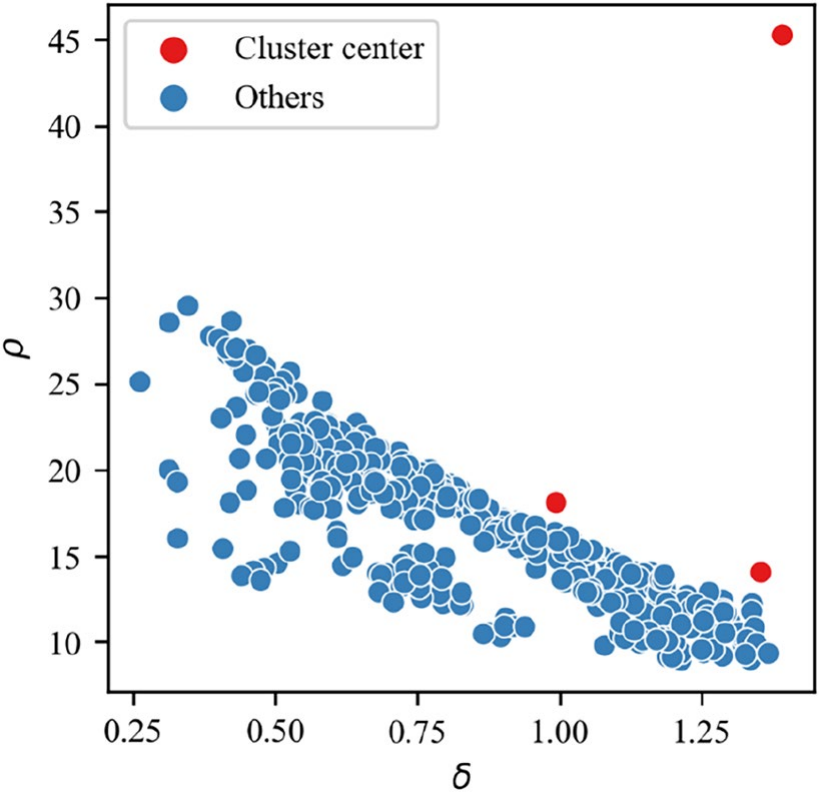
Design Map of the CFDP algorithm.

Following the steps of the CFDP algorithm in "Feature extraction" section, all test samples are plotted in the $\rho - \delta$ coordinate system to obtain the Design Map as shown in Fig. 5. Among them, the $\rho *\delta$ values of three points are significantly larger than other points, showing that there are three points with a clear advantage to become the cluster center (red points). This is the same as the number of three types of flow regimes involved in this paper. After determining them as the cluster centers, the category of the remaining points (blue) is determined in turn according to the principle in "Feature extraction" section. Finally, the number of test points corresponding to the three classifications is determined to be 120, 297, and 78 respectively.

**Figure 6:**
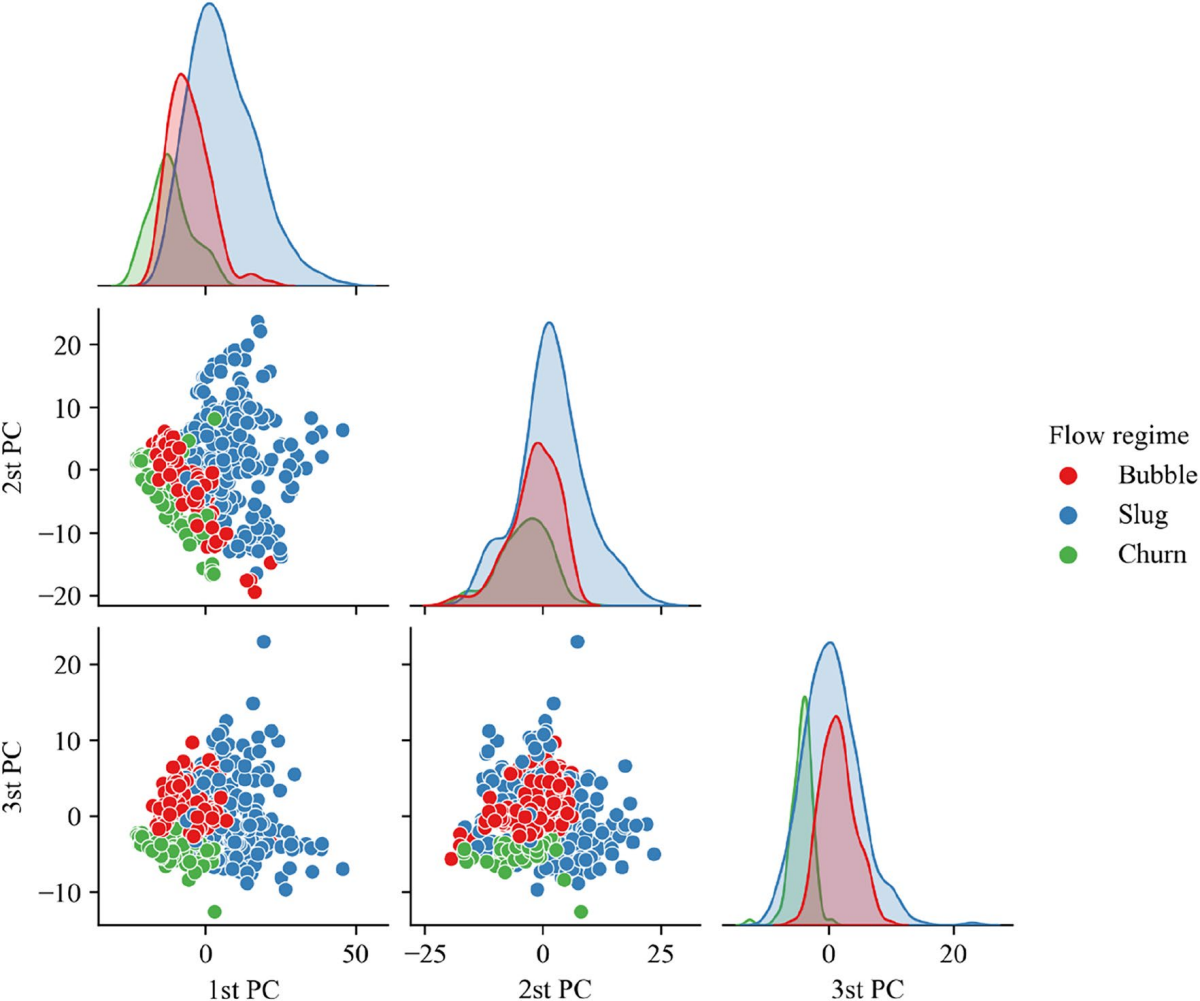
Distribution of clustering results on the first three principal components of the dimensionality-reduced space of the PSD of the DP1 signal.

**Figure 7:**
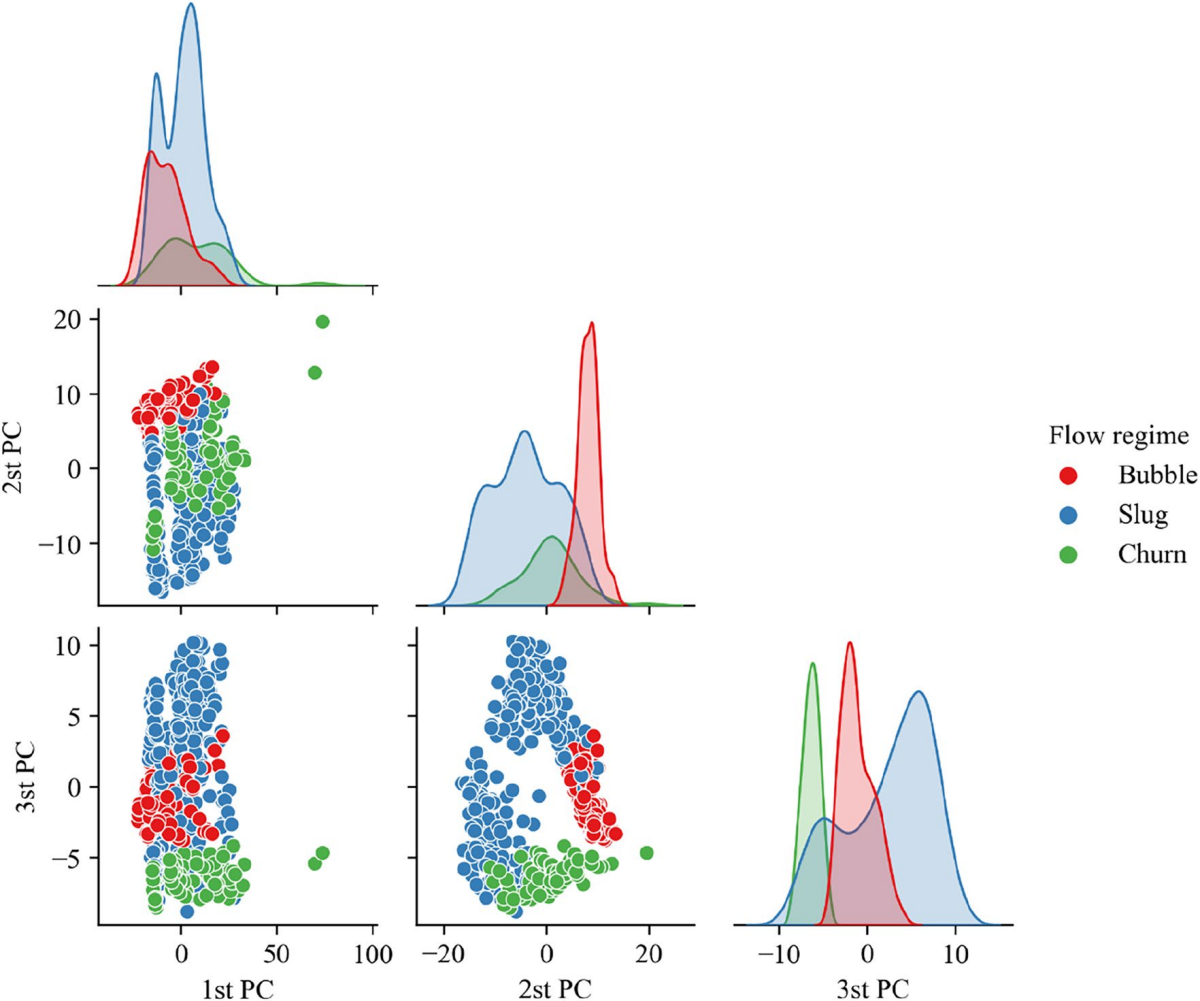
Distribution of clustering results on the first three principal components of the dimensionality-reduced space of the PSD of the DP2 signal.

Figures 6 and 7 show the distribution of the clustering results on the first three main components in the space after the PSD of the DP1 and DP2 signals is reduced. It can be seen that, firstly, whether it is on the DP1 signal or on the DP2 signal, the three categories have not been completely separated. This indicates that the three types of flow regimes cannot be distinguished using only PSD features, which verifies the necessity of adding the PDF to the features. Secondly, the distribution of clusters is obviously non-convex. This distribution makes those clustering algorithms that assume the high-dimensional spherical shape of the same cluster distribution no longer applicable. However, it can be observed that the same cluster has continuity in distribution density. This confirms the necessity of using the CFDP algorithm.

### Flow regimes identification results

**Figure 8:**
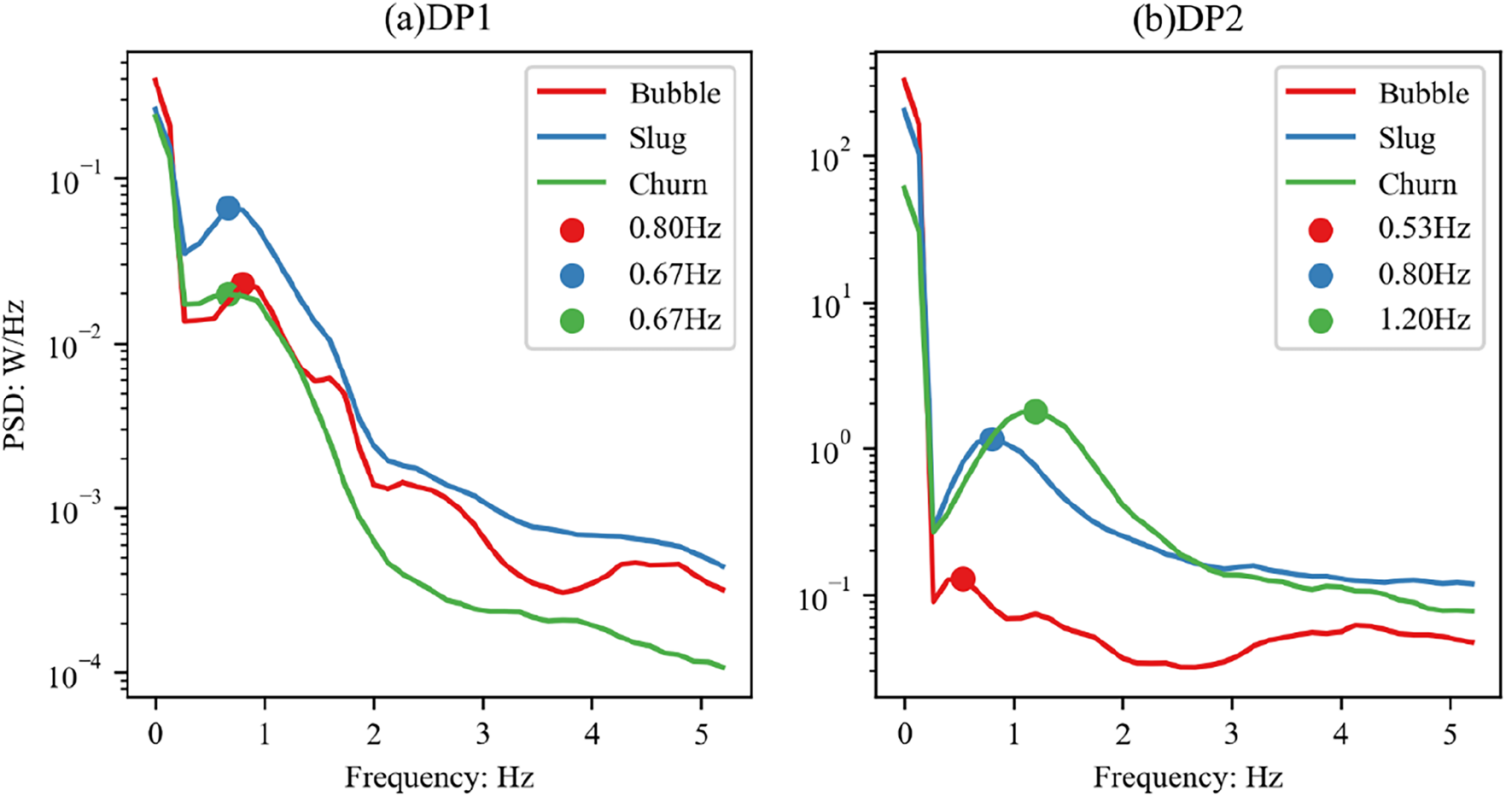
Distribution of mean PSD values of two differential pressure signals corresponding to three flow regimes.

**Figure 9:**
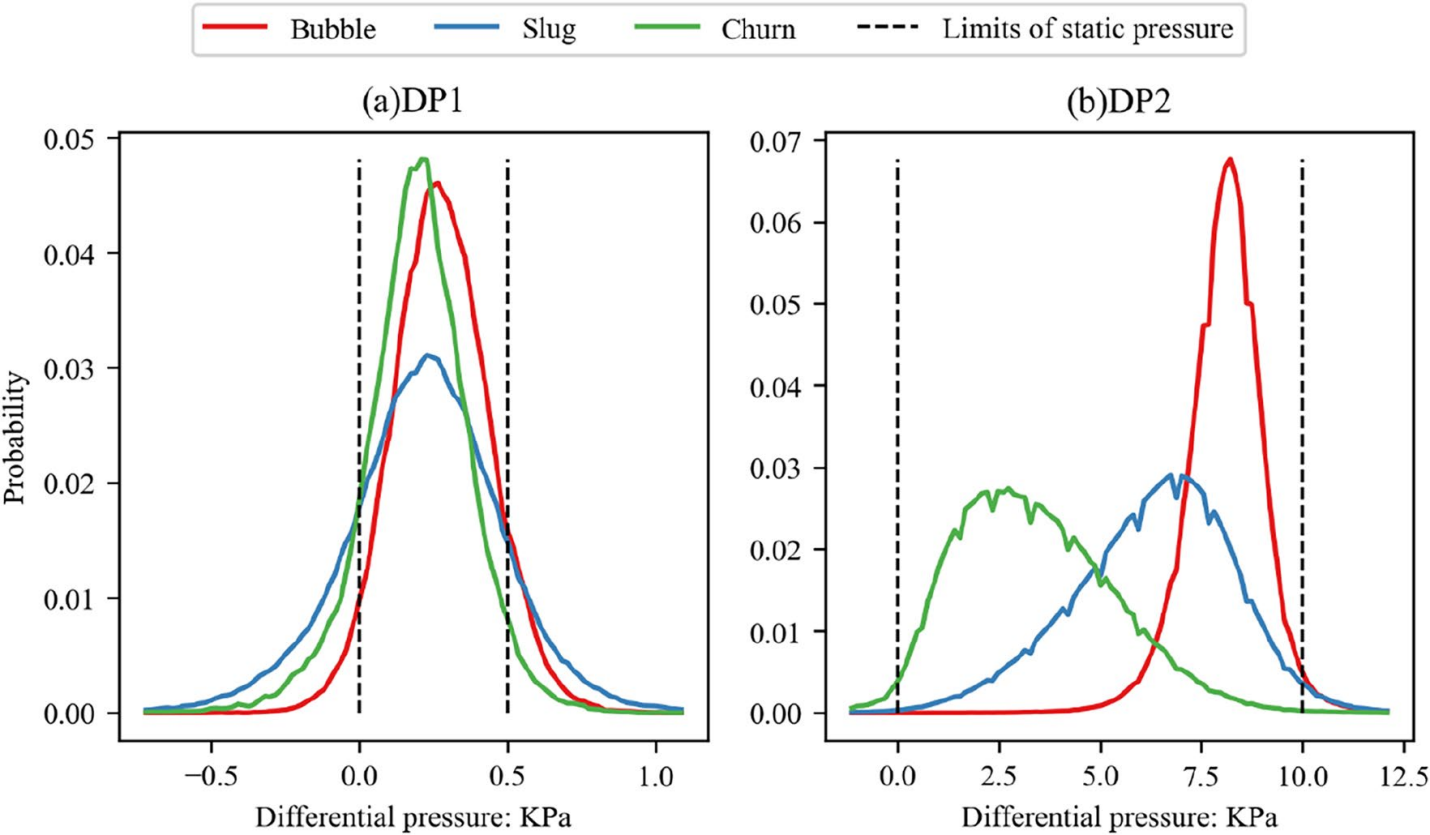
Distribution of PDF of two differential pressure signals corresponding to three flow regimes.

The three categories correspond to three flow regimes: bubbly flow, slug flow, and churn flow, respectively. Figures 8 and 9 are the average PSD and PDF plots of the two differential pressure signals corresponding to the three flow regimes.

As shown in Fig. 8, the main differences in the PSD curves corresponding to the three different flow regimes are all distributed below 5 Hz. According to the conclusions of Drahoš et al.^55^, the part below 0.5 Hz on the PSD curve is caused by environmental factors such as mechanical vibrations, and the differences in this part can be ignored. For the DP1 signal, as shown in Fig. 8a, the differences between the three different flow regimes are mainly in the 0.5–1 Hz range. In this frequency band, slug flow has the maximum power, followed by bubbly flow, and churn flow has the least power. The solid circles are the positions of the maximum values of the three curves. It can be seen that the frequencies corresponding to the maximum power of the three flow regimes are 0.8 Hz, 0.67 Hz, and 0.67 Hz, respectively.

Figure 8b presents the average of the PSD of the DP2 signals corresponding to the three different flow regimes. It can be seen that the differences between different flow regimes primarily lie in the 0.5–2 Hz range. The churn flow possesses the maximum power, followed by slug flow, and bubbly flow has the least power. The frequencies corresponding to the maximum power of the three flow regimes are 0.53 Hz, 0.8 Hz, and 1.2 Hz, respectively.

Comparing Figures (a) and (b), it can be seen that the ranking of power corresponding to three different flow regimes on two different signals is inconsistent, and the frequencies corresponding to the maximum power are not identical. The possible reason for this discrepancy is that the distance for collecting differential pressure signals by DP1 is relatively small (50 mm), which is far less than the scale of the structure of slug flow or churn flow, hence it cannot fully reflect the fluctuation characteristics of these two flow regimes.

Figure 9 displays the average PDF of the two differential pressure signals corresponding to the three different flow regimes. As can be seen in Fig. 9a, on the PDF of the DP1 signal, the curve shapes corresponding to bubbly flow and churn flow are similar, with a more concentrated distribution of differential pressure values. The primary difference lies in the mean differential pressure, whereas the curve corresponding to slug flow appears relatively flat and dispersed, indicating a larger variance in differential pressure values and suggesting strong fluctuations during the slug flow stage.

In Fig. 9b, the distribution curve forms corresponding to slug flow and churn flow are relatively similar, with a larger variance in differential pressure values, and the means exhibit significant differences. In contrast, the differential pressure values corresponding to bubbly flow are more concentrated. In the figure, the vertical black dashed lines represent the range of static water pressure corresponding to the two differential pressure signals. For DP1, this range is 0–0.5 kPa, and for DP2, the range is 0–9.8 kPa.

Comparing the two differential pressure signals, it can be observed that the areas where the DP1 signal exceeds the static pressure range are significantly more than those for DP2. This reveals that the axial differential pressure of two-phase flow in the vertical annulus has greater fluctuations at a smaller scale.

### Eccentricity effect analysis

**Figure 10:**
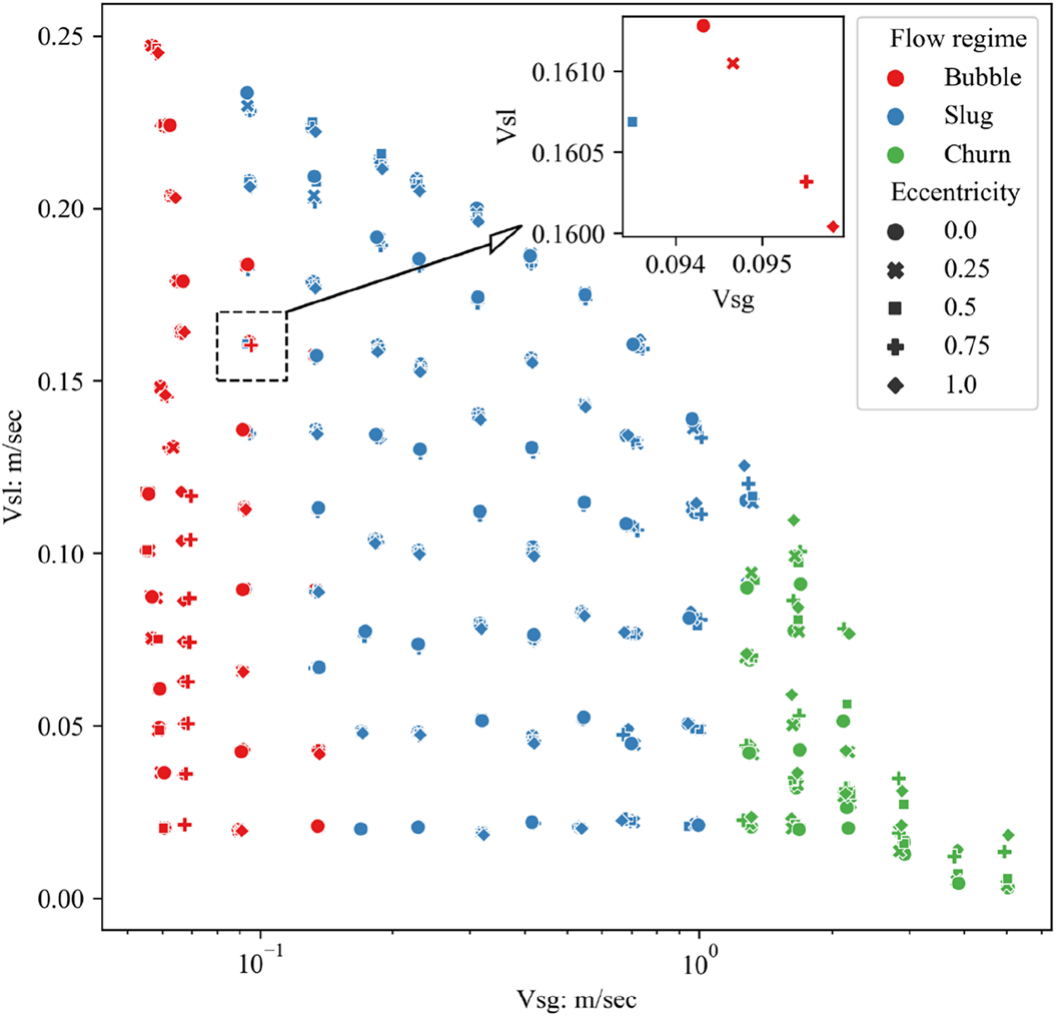
Flow regime map of 125 mm/75 mm annular gas–liquid counter-current flow. In the transition area between bubbly flow and slug flow, in some pipe columns with intermediate eccentricities, the flow transitions to slug flow first.

Figure 10 depicts the distribution map of the flow regimes for all test points, where different flow regimes are marked with different colors, and different symbols represent the eccentricity of the pipe column during the test. Here, Vsg represents the gas superficial velocity, and Vsl denotes the liquid superficial velocity.

As can be observed, in most cases, when the superficial velocities of gas and liquid are the same, the same flow regime is present under different eccentricities. However, in some flow regime transition areas, when the superficial velocity of gas and liquid are very close, different flow regimes are classified under different eccentricities. As shown in the subfigure in Fig. 10, it represents test data from five different eccentricities with similar gas–liquid flow rates. At an eccentricity of e = 0.5, although the corresponding gas superficial velocity is less than the cases of e = 0, 0.25, 0.75, and 1, its flow regime is still classified as slug flow instead of bubbly flow. In fact, in the transition area from bubbly flow to slug flow, multiple sets of test data with similar gas–liquid flow rates show that the flow regime is bubbly flow when e = 0, and it transitions to slug flow at other eccentricities.

According to the analysis results in Figs. 8 and 9, under the slug flow state, the average power of low-frequency fluctuations is higher than that of bubbly flow, and the average pressure gradient is lower than that of bubbly flow. However, the differences in the gas flow rates at each test point in the subfigure of Fig. 10 are not significant, hence the pressure gradients are similar. Therefore, the main difference lies in the power of differential pressure fluctuations.

**Figure 11:**
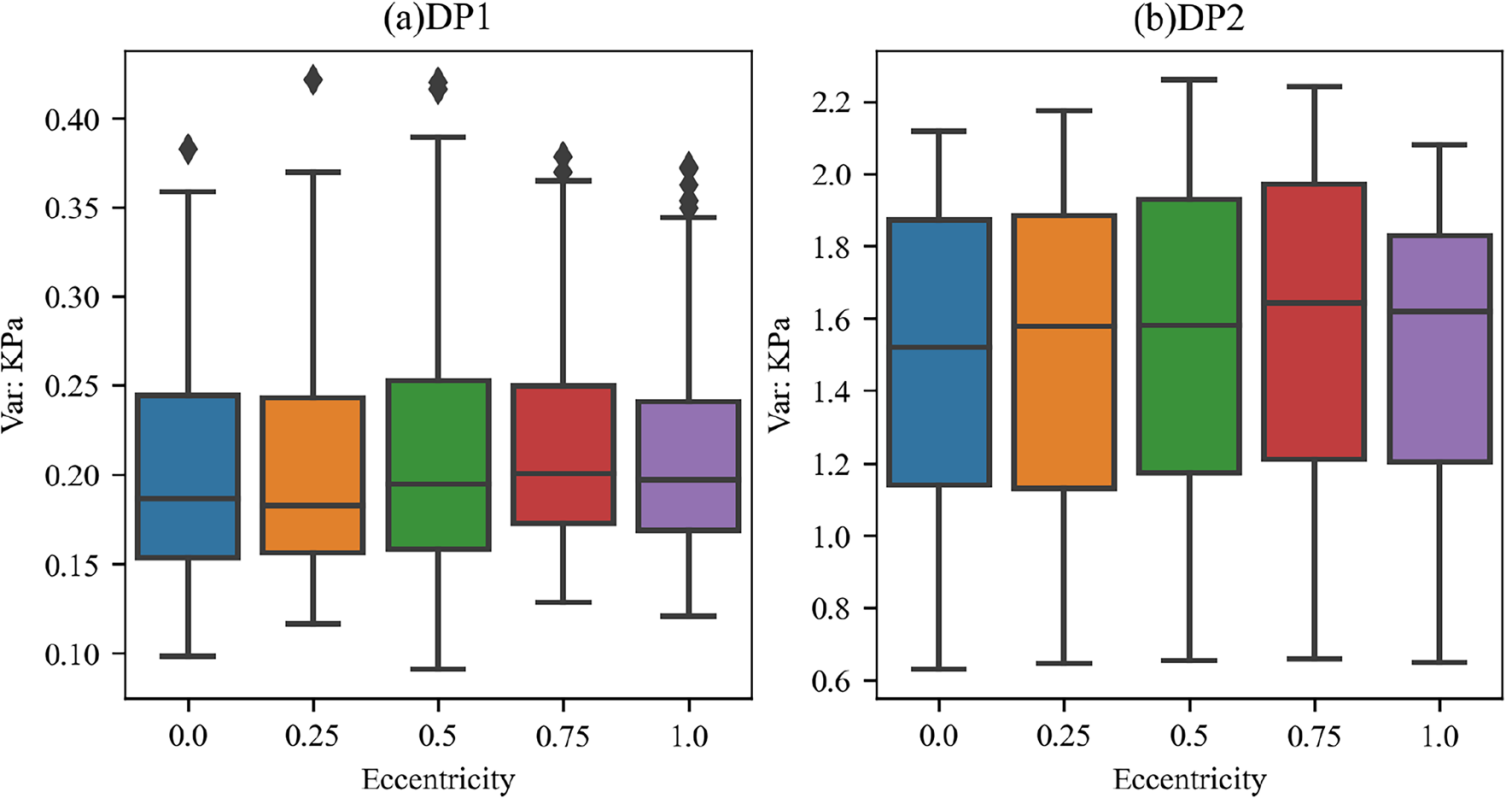
Comparison of the variance of the two differential pressure signals at different eccentricities.

To analyze the differences in differential pressure fluctuations at different eccentricities, a statistical comparison of the variance data of the two differential pressure signals at different eccentricities was performed, as shown in Fig. 11. For both DP1 and DP2 signals, the mean variances corresponding to different eccentricities are smallest when e = 0 and largest when e = 0.75. The range of variance distribution is largest when e = 0.5 and smallest when e = 1. Considering that the larger the variance of the differential pressure values, the stronger the fluctuation, the explanation for the phenomenon displayed in the bubbly flow-slug flow transition area in Fig. 10 is as follows: Although the gas–liquid flow rates are similar, the variance of the differential pressure values is larger when the eccentricity is at a medium value, i.e., e = 0.5, 0.75. Therefore, it is more similar to slug flow, which has a larger fluctuation power. When e = 0, the variance of differential pressure is the smallest, and it finally transitions to the slug flow stage. This manifestation is consistent with the conclusions of Kelessidis et al.^19^ who compared the flow regime transitions in two vertical annuli, concentric (e = 0) and semi-concentric (e = 0.5), and believed that the change in eccentricity does not greatly influence the flow regime transition. However, in the case of large eccentricity rather than small, it was observed that local flow regimes are more likely to develop at lower gas velocities.

### Comparison of flow regime clustering results and visual recognition results

**Figure 12:**
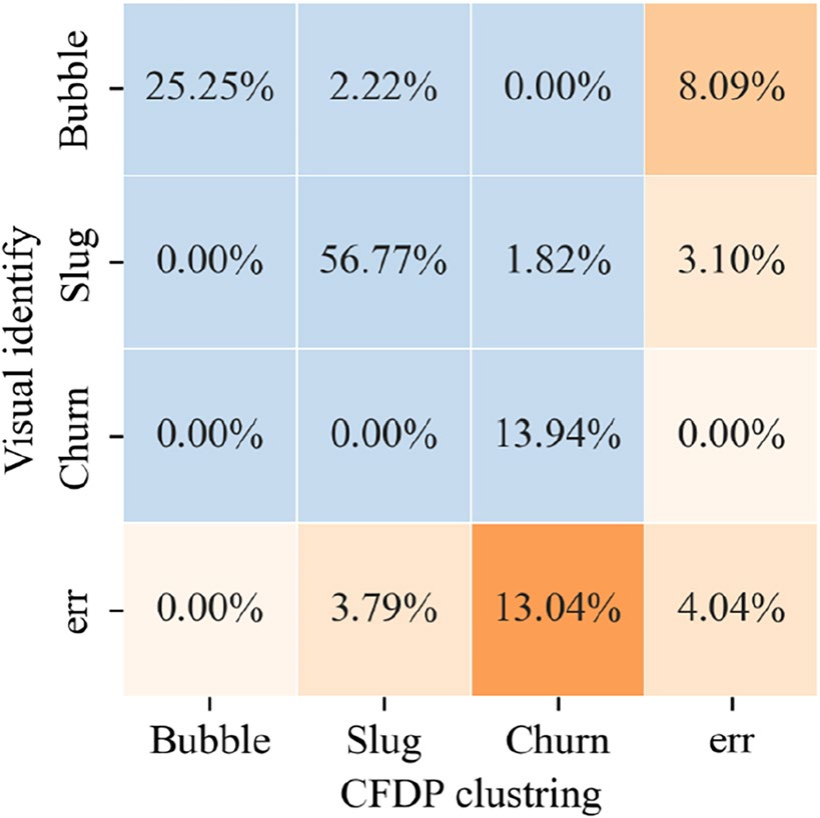
Confusion matrix of flow regime clustering results and visual recognition results.

Finally, as a test of the accuracy of flow regime recognition by this method, a comparison was made between the flow regime clustering results and visual recognition results. The confusion matrix is shown in Fig. 12. It can be seen that the cases with a large difference between the two methods are mainly: (a) The clustering method identified 13.04% of the flow regimes recognized as churn by the visual method as slug; (b) The visual method identified 8.09% of the flow regimes recognized as bubbly by the clustering method as slug. Overall, the inconsistency between the two methods accounts for 4.04% of the total, reflecting good consistency.

**Figure 13:**
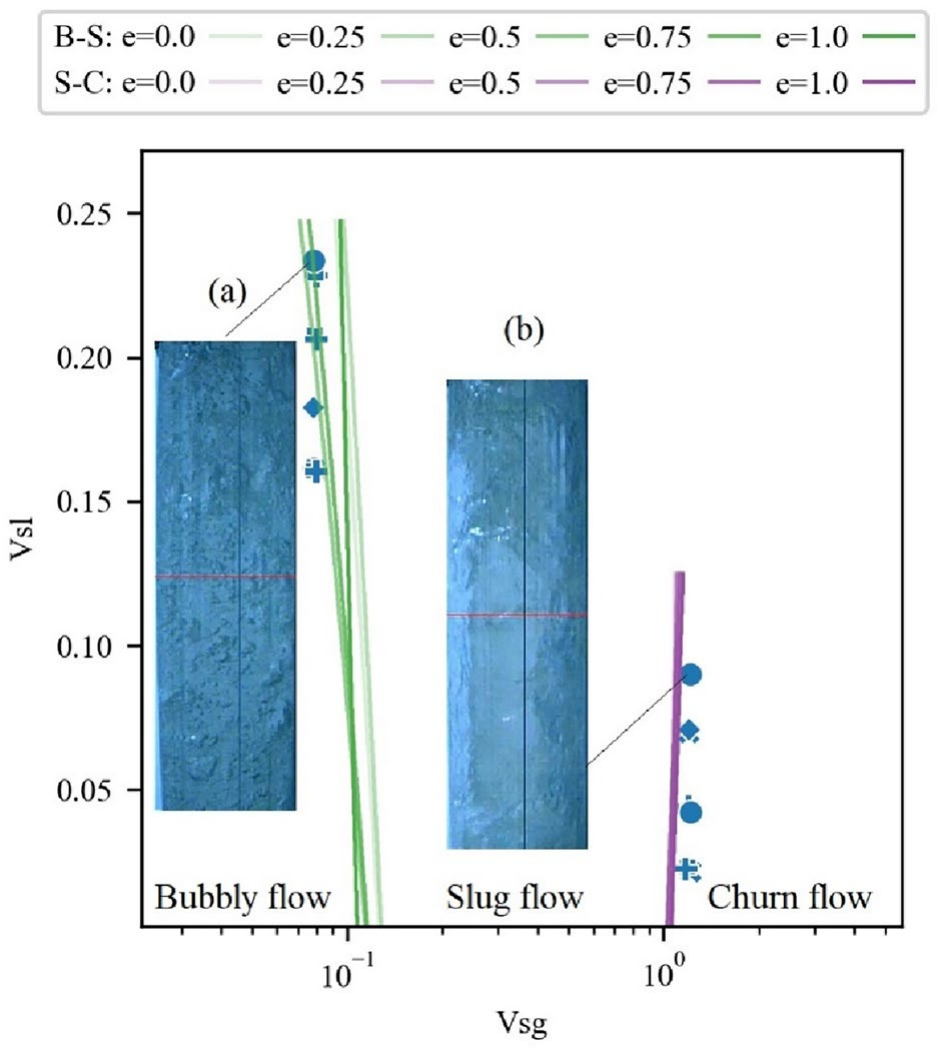
Samples with divergent flow regime identification results: (**a**) Visually recognized as "slug" but identified as "bubbly" by the clustering method (**b**) Visually recognized as "slug" but identified as "churn" by the clustering method.

To analyze the reasons for the differences in the results of the two flow regime recognition. methods, samples with different recognition results were plotted on the flow regime map. At the same time, support vector classifiers (SVC) were trained using the superficial velocities and flow regimes of the experimental data corresponding to different eccentricities to divide the areas of different flow regimes. This is shown in Fig. 13, where the green line bundles represent the transition boundary between bubbly flow and slug flow, and the purple line bundles represent the transition boundary between slug flow and churn flow. Different alphas represent different eccentricities. As can be seen, the samples with differences in recognition results are mainly located in the flow regime transition area. Subfigure (a) shows a high-speed photographic image of a sample that is visually recognized as "slug" but recognized as "bubbly" by the clustering method. Subfigure (b) shows a sample that is visually recognized as "slug" but recognized as "churn" by the clustering method.

In fact, in vertical annular gas–liquid two-phase flow with larger geometric dimensions, it is difficult for regular Taylor bubbles to appear. Therefore, flow regime classification based on visual judgment is actually not reliable, especially in the flow regime transition area. The accuracy of recognition largely depends on subjective experience and the quality of high-speed photographic images. In this case, the clustering method based on quantitative calculation should have better accuracy and objectivity.

## Conclusion

A vertical annular pipe column with adjustable eccentricity between the inner and outer pipes was designed. Gas–liquid two-phase counter-current flow experiments were conducted under different combinations of gas and liquid flow rates and eccentricities, and differential pressure data were collected at two different distances.

The PDF of the two differential pressure signals and the PSD reduced by the PCA method formed the features. The flow regimes of each experimental data were clustered using the density-based clustering algorithm CFDP, achieving an objective and consistent flow regime classification for vertical annular gas–liquid counter-current flow.

The analysis of the two differential pressure signals indicates that for the differential pressure signal DP1 collected at a smaller distance (50 mm), the differences in the PSD of different flow regimes mainly lie in the 0.5–1 Hz range. The power intensities of the three involved flow regimes are strongest in slug flow, followed by bubbly flow, and weakest in churn flow. In terms of differential pressure distribution, the differential pressure distributions of bubbly flow and churn flow are concentrated, while slug flow distribution is more dispersed. For the differential pressure signal DP2 collected at a larger distance (1000 mm), the differences in the PSD of different flow regimes mainly lie in the 0.5–2 Hz range. The power intensities of the three involved flow regimes are strongest in churn flow, followed by slug flow, and weakest in bubbly flow. In terms of differential pressure distribution, the differential pressure distribution of bubbly flow is concentrated, while the distributions of slug flow and churn flow are more dispersed.

Based on the flow regime classification results, a flow regime map was drawn, and the influence of pipe column eccentricity on the flow regime was analyzed. The results show that in most cases, the pipe eccentricity has no significant effect on the flow regime. However, in the flow regime transition area, such as the bubbly flow-slug flow transition area, when the superficial velocities of gas and liquid are not much different, some flow processes with medium eccentricity values, i.e., e = 0.5, 0.75, tend to first transition to slug flow.

A comparison was made between the visual recognition results and clustering results of the flow regime. Samples with different recognition results account for 4.04% of the total and are mainly located in the flow regime transition area, where it is usually difficult to visually identify the flow regime. Therefore, this method provides an objective classification method for annular gas–liquid two-phase flow.

## Data Availability

The data presented herein are available upon request from the project PI Cao Feng by the e-mail: feincao@qq.com.
